# Peak-at-end rule: adaptive mechanism predicts time-dependent decision weighting

**DOI:** 10.1038/s41598-020-74924-x

**Published:** 2020-10-20

**Authors:** Ryuto Yashiro, Isamu Motoyoshi

**Affiliations:** Department of Life Sciences, The University of Tokyo, 3-8-1 Komaba, Meguro-ku, Tokyo, 153-8902 Japan

**Keywords:** Neuroscience, Psychology

## Abstract

Humans make decisions under various natural circumstances, integrating multiple pieces of information that are distributed over space and time. Although psychophysical and physiological studies have investigated temporal dynamics underlying perceptual decision making, weighting profiles for inliers and outliers during temporal integration have yet to be fully investigated in most studies. Here, we examined the temporal weighting profile of a computational model characterized by a leaky integrator of sensory evidence. As a corollary of its leaky nature, the model predicts the recency effect and overweights outlying elements around the end of the stream. Moreover, we found that the model underweights outlying values occurring earlier in the stream (i.e., robust averaging). We also show that human observers exhibit exactly the same weighting profile in an average estimation task. These findings suggest that the adaptive decision process in the brain results in the time-dependent decision weighting, the “peak-at-end” rule, rather than the peak-end rule in behavioral economics.

## Introduction

We humans make decisions ranging from purchasing a product that fits our preferences to choosing the best career in the face of future uncertainty. These behaviors can be reduced to a decision-making framework in which we choose one specific option among many. Classical and behavioral economics studies have provided detailed descriptions of optimal and irrational decisions^1–5^. One typical example is the peak-end rule whereby humans’ retrospective judgments on their experience are disproportionately influenced by its most emotionally or physically intense moments and its end^6–8^. However, the computational process that gives rise to such cognitive biases has been overlooked for decades.

In contrast, a number of psychophysical and physiological studies have determined the computational mechanisms underlying perceptual decision making in simple laboratory settings^9–11^. Despite the differences in experimental paradigms among those studies, they have commonly uncovered a basic mechanism of integrating multiple pieces of information during perceptual decision making. Importantly, most studies have argued that humans integrate sensory evidence for decisions in an adaptive manner.

Researchers have been working on determining the characteristics of spatial integration independently from temporal integration. For instance, when humans integrate sensory signals from multimodal sources, they adopt the maximum likelihood estimation (MLE) rule, assigning optimal weights to multiple stimuli according to their different reliability levels^12,13^. Similarly, studies on texture perception have suggested a mechanism of limited sampling for estimation of average orientation, rather than simultaneous averaging of all elements across space^14–16^. In line with these findings, recent studies of estimation of average color and orientation have shown that humans exhibit a robust averaging strategy by which outliers are excluded, and in turn, greater weight is given to trustworthy inliers^17,18^. This strategy is consistent with perception and sensorimotor control, which follow optimal Bayesian theory^19,20^, and it seems adaptive, as it paradoxically enhances the performance of perceptual decision making even with a large amount of internal noise^18^.

On the other hand, a number of psychophysical studies have constructed various computational models for temporal integration^21–26^, most of which are virtually equivalent to the Drift Diffusion Model (DDM)^27^. The typical version of DDM is comprised of a random-walk process that accumulates sensory evidence over time and two bounds that set a quantitative criterion for a decision. It successfully accounts for skewed reaction time distributions and the speed/accuracy tradeoff^28,29^. Additionally, physiological studies have identified neural activities that correspond to the temporal dynamics of DDM^30–32^.

However, such computational models (including DDM) are far from complete because they assume the accumulation of absolute signal values regardless of the temporal span. One possible alternative to such time-independent models is the integration of decision variables with leakage over time^24,33^. Also, recent work offers a computational account for temporal integration: sensory inputs are encoded relative to the local context by adjusting the gain of the inputs along with linear integration^34^. The core mechanism of these time-dependent models is leaky integration, in which a decision depends on the sum of successive sensory signals multiplied by decision weights that exponentially decay over time. It should be noted that leaky integration replicates the recency effect widely observed in studies of perceptual decision making, whereby samples presented later wield greater influence over decisions^9,24,34–37^, providing further support for the time-dependent leaky decision process.

In view of these findings on weighting profiles (robust averaging and the recency effect) accompanying the computational mechanisms, it can be hypothesized that inlying and later information has a greater influence on human decisions during temporal integration. However, no study has directly corroborated this hypothesis because of the lack of corresponding analyses between the spatial and temporal domains. More importantly, this hypothesis contrasts with the peak-end rule in behavioral economics studies in terms of the amount of weight given to outliers. The question thus arises of how humans give weight to inliers and outliers during temporal integration for perceptual decisions.

In this paper, we used theoretical and experimental examinations to elucidate a temporal weighting profile for outliers, a matter that has been overlooked in previous studies. We simulated the weighting profiles of the simple leaky integration model applied to a typical perceptual decision task. We also conducted the same task with human observers and compared the human weighting profiles with those of the model. The results show that humans underweighted outliers presented earlier in a stimulus stream (i.e., robust averaging), but overweighted those presented later in the stream. These findings manifest the time-dependent decision weighting profile (“peak-at-end” rule) in humans, as predicted by the leaky integration model.

## Results

### Analysis of weighting profiles

To examine a weighting profile generated by the decision model, we simulated response data for a simple psychophysical task in which an observer is presented with a sequence of eight Gabor patterns and decides whether the temporal average of the orientation is tilted clockwise or counterclockwise. We first generated stimulus values for 10,000 trials according to the actual distribution used in our psychophysical experiment, namely a normal distribution with a randomly set mean (− 3 to 3 deg, in steps of 0.5 deg) and SD (8 deg) for each trial.

Different aspects of the weighting profile for the simulated data can be revealed by means of two distinct analyses. One of the analyses was logistic reverse correlation, in which a multiple logistic regression model was created with orientation values in eight temporal frames and responses as independent and dependent variables, and logistic regression coefficients were calculated. The coefficients reflect how much impact the orientation in each temporal frame has on the observer’s response. This analysis thus reveals the temporal weighting bias caused by the decision mechanism (e.g., recency effect).

**Figure 1:**
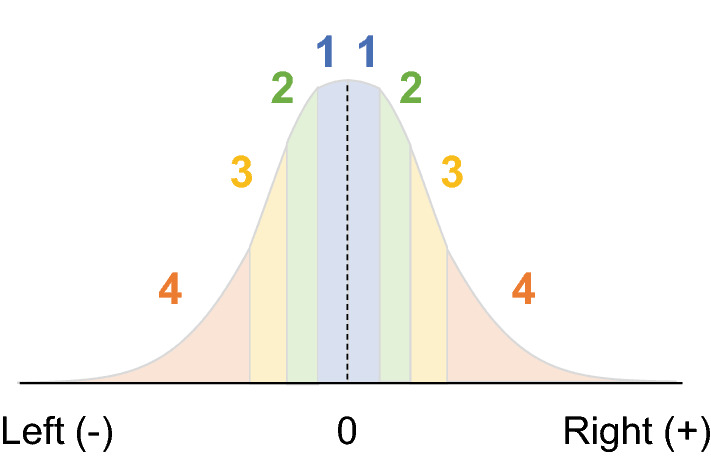
The criterion for the ranking. Each rank (1, 2, 3, and 4) represented by differently colored area comprises an equal amount of the probability mass (25% for each).

Because the present study sought to determine how the model assigns weights to inliers and outliers during temporal integration, we also conducted the following analysis. First, the orientation values across all temporal frames and trials were ranked into four categories according to how much they were tilted. The least tilted 25% were classified as rank 1, the next 25% as rank 2, the next 25% as rank 3, and the most tilted 25% as rank 4. Figure 1 shows the criterion for this ranking, which ensures that the same number of samples is included in each rank. Then, we extracted orientations with a particular rank and temporal frame to calculate how consistent the sign of the orientations and the responses were across all trials, which we call the consistency rate. A higher consistency rate for elements with a particular rank and temporal frame corresponds to overweighting of those elements. We calculated the consistency rates for orientations across four ranks and eight temporal frames, and obtained 32 consistency rates in total, allowing us to produce a weighting profile for inliers and outliers in the stimulus stream.

### An ideal observer

We first assumed an ideal observer model that integrates information evenly across all temporal frames. In this model, a decision is made based on the sign of the summed orientations over all temporal frames (Eq. 1). We obtained the weighting profiles of the model using the above analyses.

1 $$S=\sum_{t=1}^8 \theta \left(t\right)$$

**Figure 2:**
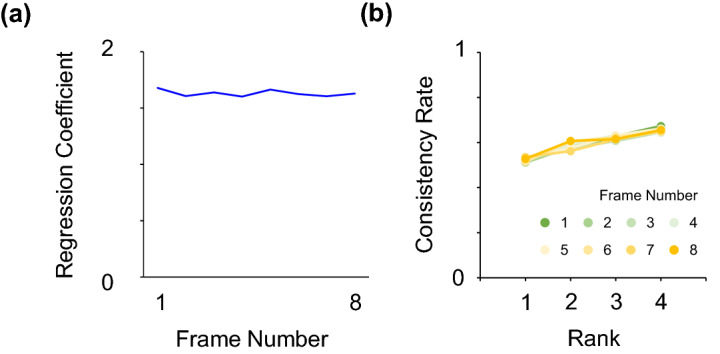
Simulated weighting profiles of the ideal observer model. (**a**) Regression coefficients across temporal frames for the ideal observer. (**b**) Consistency rates of the ideal observer for each rank and temporal frame. The eight lines representing different results for each temporal frame overlap each other.

As expected, the model predicted almost the same regression coefficients across all temporal frames (Fig. 2a), confirming that each element of information has an equal impact on the response. Figure 2b shows the consistency rates for each rank and temporal frame. Different colors represent different results for each temporal frame, but they overlap with each other. The model appears to assign greater weight to outliers (i.e., elements with a higher rank) than to inliers, as the consistency rate was higher for elements with higher ranks — a somewhat unexpected result for the unbiased ideal observer. However, this tendency is simply an artifact of the higher correlation between higher-ranked stimulus values and the temporal average. Henceforth, to reveal the sheer weighting profile for outliers, we thus calculate the difference in consistency rates between the ideal observer and the model (or human data) rather than relying on single consistency rates.

**Figure 3:**
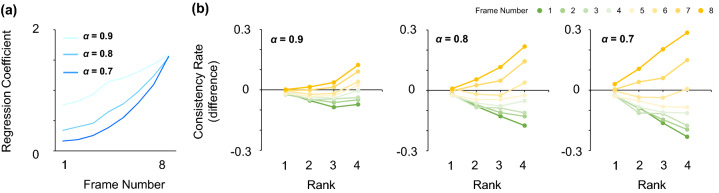
Simulated weighting profiles of the leaky integration model. (**a**) Regression coefficients across temporal frames for the leaky integration model. Different colors represent the results for different $\alpha$ values. (**b**) Consistency rates of the model for each rank and temporal frame. Results for different $\alpha$ values are shown in each panel.

### A leaky integration model

Next, we applied the two analyses to the response data of the leaky integration model. In the model, a final decision is made based on the sign of the summation of orientation signals multiplied by a weight that exponentially decays over time with additive internal noise $\varepsilon$ (Eq. 2). As mentioned earlier, this simple decay model captures the essence of prevailing models of perceptual decision making^9,24,33–37^.

2 $$S=\sum_{t=1}^8{\alpha }^{8-t} \theta \left(t\right)+\varepsilon$$

In line with previous studies^34,35^, reverse correlation analysis showed a clear recency effect: the regression coefficient was higher for later inputs, and this effect became more pronounced at decreased $\alpha$ values (Fig. 3a). We also calculated the difference in consistency rates between the model and the ideal observer for each rank and temporal frame, to probe how the model weights outlying elements (Fig. 3b). The model predicted lower consistency rates for elements with higher ranks that occur earlier in the stimulus stream. However, we found the opposite result for elements that occur later in the stream: their consistency rate increased in proportion to their rank. That is, the model downweights outlying elements that occur earlier in the sequence (i.e., robust averaging), but in turn overweights those occurring later in the sequence. These results lead to the critical notion that the model predicts a time-dependent weighting profile for outliers as a corollary of its leaky nature.

### A psychophysical experiment with human observers

In the previous section, we found that the leaky integration model exhibits a time-dependent weighting profile for outliers and a recency effect during temporal average estimation. To test whether this is also the case for humans, we probed human observers’ weighting profiles for outlying elements at different temporal locations by means of a psychophysical task and analyses identical to those used in the simulation.

**Figure 4:**
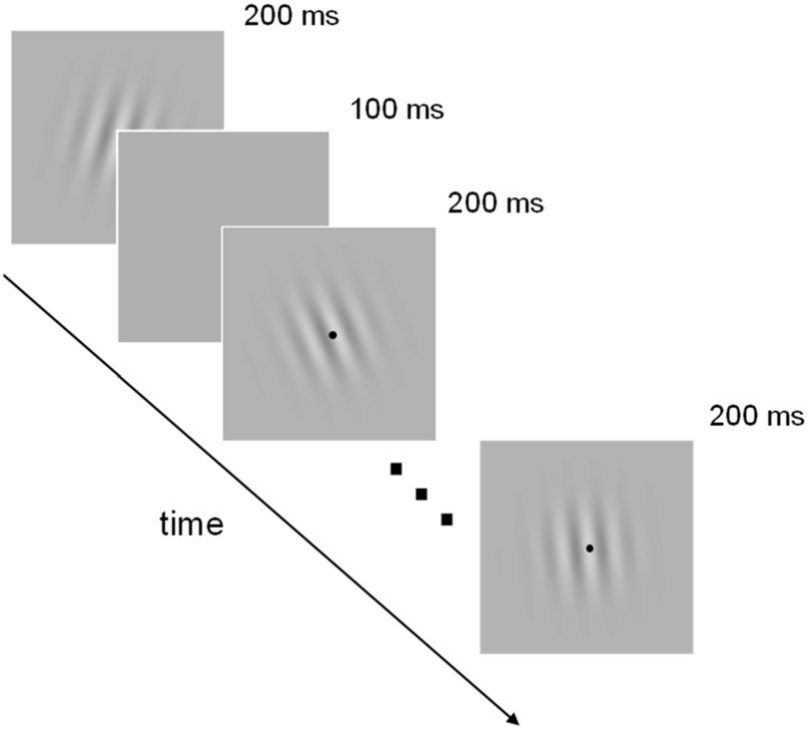
Visual stimulus used in the experiment.

One of the authors and nine naïve observers participated in the task, viewing eight successive Gabor patterns (Fig. 4). The orientation of these patterns was determined according to a normal distribution with a random mean (− 3 to 3 deg, in steps of 0.5 deg) and SD (8 or 16 deg) for every trial. Observers reported whether the temporally averaged orientation of the patterns was tilted clockwise or counterclockwise by button press (see Methods for details). Using the stimulus and response data, we performed the same analyses as we did on the simulation data to examine human observers’ weighting profiles during the task.

**Figure 5:**
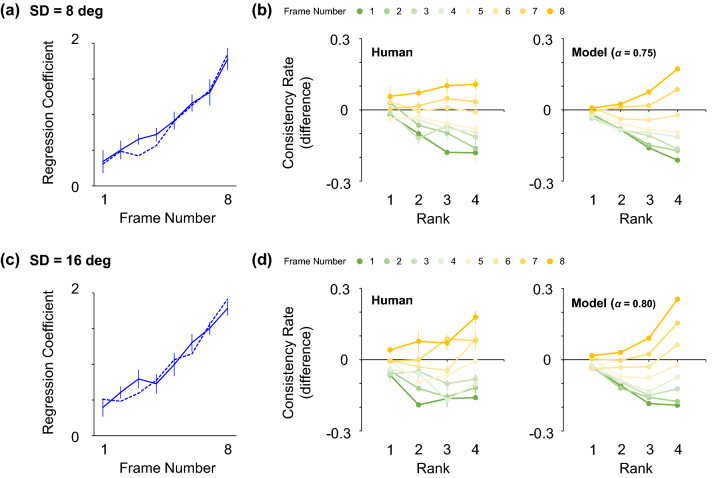
Weighting profiles for human observers. The upper and lower panels are results for different SDs of the stimulus orientation. (**a**, **c**) Regression coefficients across temporal frames for human observers. Dashed lines represent the simulated coefficients of the model using the best-fitting parameters. (**b**, **d**) Differences in consistency rates between human observers and the ideal observer for each rank and temporal frame (left panels). Simulated consistency rates of the model using the best-fitting parameters (right panels). All the conventions are the same as in Figs. 2 and 3.

We first performed reverse correlation analysis to elucidate how the observers assigned weights to information presented at each temporal frame. A clear recency effect was observed regardless of the SD of the normal distribution that generated the stimulus values (Fig. 5a,c). A two-tailed t test showed a significant difference between the average of regression coefficients for earlier (1st–4th frames) inputs and later (5th–8th frames) inputs (*t*(9) = 6.69, *p* < 0.001 [SD = 8 deg]; *t*(9) = 8.78, *p* < 0.001 [SD = 16 deg]). Then, we fitted the leaky integration model to the observed regression coefficients by optimizing the two free parameters $\alpha$ and $\varepsilon$ (SD of the additive internal noise) so that the mean squared error between the observed and predicted regression coefficients was minimized for each observer, and in this manner obtained the best-fitting parameters ($\alpha$=0.75 [s.e. = 0.04 across observers], $\varepsilon$=2.81 [0.19] when SD = 8 deg; $\alpha$=0.80 [0.04], $\varepsilon$=4.76 [0.12] when SD = 16 deg). The simulated regression coefficients were almost identical to those of the human observers (dashed lines in Fig. 5a,c).

We also ranked orientations across all trials and temporal frames in accordance with the criterion introduced in the simulation section (Fig. 1), and we calculated consistency rates for each rank and temporal frame. The left panels in Fig. 5b,d show the differences in consistency rates between human observers and the ideal observer. The observed human consistency rates bore a qualitative resemblance to those of the model with the best-fitting parameters (right panels in Fig. 5b,d). This suggests that humans discounted outliers occurring earlier in the stream (robust averaging) but overweighted those occurring later in the stream, as predicted by the leaky integration model. A significant interaction was revealed by a two-way repeated measures ANOVA with rank and temporal frame as factors (*F*(21, 189) = 2.00, *p* = 0.007 [SD = 8 deg]; *F*(21, 189) = 2.62, *p* < 0.001 [SD = 16 deg]). These results suggest that humans exhibit a time-dependent weighting profile to outliers as well as a recency effect, both of which are traced to the leaky integration mechanism.

Psychophysical evidence shows that the neural coding of orientation is uncertain and biased toward the cardinal orientations, i.e., vertical in the case of our stimuli^38,39^. This bias would potentially give rise to robust averaging as inliers in our experimental setting correspond to the vertical orientation. We confirmed that this is true by using a leaky integration model with orientation inputs biased toward the vertical. However, the model failed to replicate the overweighting of outliers in the last few frames, precluding the orientation-dependent uncertainty as a main source for the time-dependent weighting policy.

## Discussion

Through a model simulation and a psychophysical experiment with human observers, the present study offers insights into temporal weighting profiles during evidence accumulation for perceptual decision making. The leaky integration mechanism predicts not only the recency effect but also a characteristic time-dependent weighting profile: less weight is given to outlying than inlying elements occurring earlier in a stream (i.e., robust averaging), whereas greater weight is given to outliers occurring later in a stream. Importantly, human observers in the typical average estimation task adopted the time-dependent weighting policy predicted by the model. These facts corroborate the presence of the leaky integration mechanism in perceptual decision making, in line with previous studies^24,33^.

Although robust averaging is observed while integrating multiple pieces of evidence that are distributed over space^17,18^, the present study indicates that humans also exhibit such a weighting policy while integrating temporal evidence. This result implies the commonality of the characteristics underlying evidence integration between the spatial and temporal domains, consistent with some studies which have posited sequential sampling even for integration of spatially distributed information that is constant over time, rather than parallel processing across space^40,41^. This assumption seems reasonable, as humans in the environment sample different local information multiple times per second with saccades, even though the source may remain invariant over time^42^.

The recency effect and overweighting of outliers we observed in the present study are reminiscent of the peak-end rule. It is intriguing that humans use temporal information to make decisions similarly across different tasks (perceptual and economic decision making), although those tasks are distinct in many respects. First, there is a fundamental difference in terms of decision type: perceptual judgment for sensory stimuli vs. emotional evaluation for an event or experience. Another difference lies in the timescale of temporal integration: an observer typically integrates evidence across a few seconds at most in a perceptual decision task, but integration can take place over minutes or even a few hours in an economic decision task. Although these differences raise the question of why similar weighting profiles are obtained across domains, subjects actually make judgments on the basis of memory within a few seconds even in economic decision tasks with long integration periods. Further, some studies have proposed the hypothesis that memory retrieval might involve sequential sampling of momentary evidence, as with perceptual decisions^41,43^. Taken together, the recency effect and overweighting of outliers might be ubiquitous across different types of judgments that involve sequential sampling of multiple pieces of information, whether they derive from sensory stimuli or memory.

It is noteworthy that no behavioral economics study has provided evidence for robust averaging that contrasts with the peak-end rule, indicating that robust averaging is uniquely observed in perceptual decision making. Here, we raise the possibility that robust averaging could also be observed in economic decision tasks. Previous behavioral economics studies illustrated the peak-end rule by showing significantly high partial regression or correlation coefficients between peak values during events and subjects’ retrospective evaluations of their experiences^6,7^. These observations are insufficient to support the peak-end rule because there is an inherently high correlation between a peak value and the overall average of sequential data^44^, thus hindering reliable conclusions about the peak-end rule. In fact, some studies have reported conflicting results that the average score is better than the peak score as a predictor of overall evaluations of experiences^45,46^. In any case, simply calculating the correlation between a single value extracted from an overall dataset and a retrospective value is insufficient to validate the peak-end rule. If the same analyses as we performed in the present study were conducted in behavioral economics studies, the same time-dependent weighting profile, including robust averaging, could be observed even in those studies. In other words, humans’ retrospective judgment about their experience may be influenced only by peak values that occur later in the course of an event, but not by those occurring earlier in the course of an event, which we call “peak-at-end” rule. In sum, more rigorous quantitative analysis has the potential to reveal robust averaging, even in economic decisions.

The present study indicates that robust averaging and the peak-at-end rule are natural consequences of the leaky temporal integration process where sensory evidence in each temporal frame is multiplied by an exponentially decaying weight. However, this mathematical description of the decision process does not tell us anything about the computational mechanisms in the brain that elicit the characteristic biases. One possible mechanism is integration with gain control, where sensory inputs are encoded relative to the local context by adjusting the gain of the inputs^34,47^. This decision mechanism is consistent with the fact that neurons in lower-level visual systems adjust their sensitivity to a wide range of light intensities^48,49^ and modulate their responses depending on the summed activity of pools of neurons^50,51^. If we adopt this framework, the observed biases can be qualitatively explained as follows: the process involves adaptation to a sudden change, protecting decisions from noise (i.e., outliers) that might cause false judgments^34,35^. However, this process does not hold true when a sudden change occurs around the end of the integration, because the observer ends up making a decision before the effects of the outliers are fully attenuated, thereby resulting in outliers occurring around the stimulus offset having a greater influence on a decision. Temporal integration with gain control thus predicts not only robust averaging, which is an optimal policy in the presence of heavy internal noise^18^, but also the suboptimal peak-at-end rule as a tradeoff for its adaptive property.

While the leaky integration model captures all aspects of the present study’s human data, it remains unknown whether it also underlies decision making for other attributes such as color and shape or tasks with various timescales of evidence accumulation. Nevertheless, as the model naturally predicts tendencies that have been widely observed in distinct research domains from psychophysics to behavioral economics, the present findings underline the possibility that leaky temporal integration (or possibly, integration with gain control) is a general computational mechanism for decision making. Our study opens the path for further investigation of the computational mechanisms that commonly underlie both weighting policies in perceptual decision making and cognitive biases in economic decision making.

## Methods: psychophysical experiment

### Observers

One of the authors and nine naïve paid volunteers with corrected-to-normal vision, participated in the experiment. All experiments were conducted with a permission from the Ethics Committee of the University of Tokyo with written informed consent taken from all the participants, and the Declaration of Helsinki guidelines were followed.

### Apparatus

Visual stimuli were generated by a graphics card controlled by a PC and displayed on a LCD monitor (BenQ XL2430T) which had a pixel resolution of 0.02 deg/pixel at a viewing distance of 100 cm we used^35^. The refresh rate was 60 Hz. The mean luminance of the uniform background was 69.0 cd/m^2^. All experiments were conducted in a dark room.

### Stimuli

Stimulus was eight frames of Gabor patterns that were presented one after the other in the center of the screen (Fig. 4). Each frame was presented for 200 ms followed by a uniform blank of 100 ms. Gabor pattern had a carrier spatial frequency of 1.56 c/deg and a Gaussian window with a SD of 5.12 deg. The Michelson contrast was 0.4. The mean luminance was equal with the uniform background. The orientation of Gabor pattern at each frame was determined according to a normal distribution with a specific mean and SD^35^. The mean across frames was randomly decided from − 3 to + 3 deg with an equal step of 0.5 deg, and the SD was 8 or 16 deg. A black fixation dot was presented in the center of the screen throughout the experiment.

### Procedure

On each trial, observers viewed the stimulus binocularly and indicated whether the temporal average of the Gabor orientation was tilted left or right by button press^35^. Observers were instructed to fixate on the fixation point and to respond within 0.5 s after the stimulus offset. If the observers’ response exceeded the deadline, auditory feedback was given, and the data on those trials were excluded from the analyses^35^. Each observer completed at least three sessions for a total of 270 trials.

### Data analysis

For the data of each observer, the same set of analyses, i.e., the reverse correlation analysis and rank analysis that were performed on the models' data (see Results section in detail), were conducted to obtain the weighting profile. The weighting data were averaged across observers. A two-tailed t test was conducted as a significant proof of the recency effect. To see if weighting profiles for outliers are significantly different across temporal frames, we also performed a two-way repeated measures ANOVA with the rank and temporal frame as factors.

## Data Availability

The datasets generated and analyzed during the current study are available from the corresponding author on reasonable request.
